# Both *Matricaria chamomilla* and Metformin Extract Improved the Function and Histological Structure of Thyroid Gland in Polycystic Ovary Syndrome Rats through Antioxidant Mechanism

**DOI:** 10.3390/biom10010088

**Published:** 2020-01-05

**Authors:** Ahlam Abdulaziz Alahmadi, Areej Ali Alzahrani, Soad Shaker Ali, Bassam Abdulaziz Alahmadi, Rana Ali Arab, Nagla Abd El-Aziz El-Shitany

**Affiliations:** 1 Department of Biological Sciences, College of Science, King Abdulaziz University, Jeddah 21589, Saudi Arabia; aahmadi1000@hotmail.com (A.A.A.); aalzahrani2278@stu.kau.edu.sa (A.A.A.); 2 Department of Anatomy, Cytology, and Histology, College of Medicine, King Abdulaziz University, Jeddah 21589, Saudi Arabia; soadshaker@gmail.com; 3 Department of Histology, College of Medicine, Assiut University, Assiut 71515, Egypt; 4 Department of Biology, College of Science, Taibah University, Madinah 42353, Saudi Arabia; bahmadi@taibahu.edu.sa; 5 Medicine Program, Ibn Sina National College for Medical Studies, Jeddah 22421, Saudi Arabia; charmingpearl1996@hotmail.com; 6 Department of Pharmacology and Toxicology, College of Pharmacy, King Abdulaziz University, Jeddah 21589, Saudi Arabia; 7 Department of Pharmacology and Toxicology, College of Pharmacy, Tanta University, Tanta 31527, Egypt

**Keywords:** *Matricaria chamomilla*, PCOS, hypothyroidism, oxidative stress, apoptosis, estrogen, histopathology

## Abstract

There is increasing proof that polycystic ovary syndrome (PCOS) is associated with the increased frequency of thyroid disturbances. Chamomile (*Matricaria chamomilla* L.) herb and metformin showed therapeutic efficacy against polycystic ovary syndrome (PCOS). This study aimed to investigate the possible therapeutic effect of both chamomile flower extract and metformin against thyroid damage associated with PCOS in rats. The PCOS model was developed in rats by injecting estradiol valerate, and it was confirmed to be associated with thyroid hypofunction biochemically and pathologically. Treatment of PCOS rats with both chamomile extract and metformin resulted in an improvement in serum level of thyroid hormones (TSH, *p* < 0.01; T3 and T4, *p* < 0.05) and the disappearance of most thyroid gland pathological changes demonstrated by light and electron microscopes. They also reduced the level of serum estrogen (*p* < 0.01). Both chamomile extract and metformin decreased MDA (*p* < 0.05) and increased GPx and CAT (*p* < 0.01). Only chamomile extract increased GSH (*p* < 0.01). Both treatments reduced the apoptotic death of thyroid cells as noted by the reduction of caspase-3 immunoexpression (*p* < 0.01). In conclusion, both *Matricaria* *chamomilla* extract and metformin ameliorated hypothyroidism associated with PCOS through an antioxidant and antiapoptotic mechanism.

## 1. Introduction

Subclinical hypothyroidism (SCH), which may be interpreted as an increased thyroid-stimulating hormone (TSH) level together with normal thyroxine (T4) level and absence of obvious symptoms of hypothyroidism is often more prevalent than overt hypothyroidism [1]. Despite the fact that SCH is a mild type of hypothyroidism, it often results in anovulatory cycles, imbalances in sex hormones, subfertility, and detrimental pregnancy outcomes [2,3,4]. Moreover, females with SCH face an increase in the metabolic danger of obesity, insulin resistance, and hyperlipidemia [5,6].

Polycystic ovarian syndrome (PCOS) is among the most prevalent endocrine diseases as it affects 15% to 20% of ladies at reproductive age. The syndrome is characterized mainly by anovulation and hyperandrogenism [7]. Ladies with the PCOS are at high risk of developing a variety of metabolic syndrome and endocrinological disorders, including obesity, resistance to insulin, subfertility, and miscarriage [8,9]. Furthermore, there is growing evidence to indicate that PCOS is related to the increased incidence of thyroid disorders, including nodular goiter and autoimmune thyroiditis [10].

Assuming that women with PCOS and SCH share nearly the same features, it can be deduced that the occurrence of PCOS may be correlated with SCH’s initiation and progress [9]. Our research group recently reported an experimental model of estradiol valerate-induced PCOS in female Wistar rats that is accompanied by thyroid gland dysfunction [11]. Estradiol valerate is a long-acting estrogen used for anovulatory PCOS induction. This agent interferes with the excretion of hypothalamic gonadotropin releasing hormone, consequently, it impedes both luteinizing hormone (LH) secretion and storage [12]. The physiology and histology of the ovary in estradiol induced PCOS model are like those of the human ovary [13].

Chamomile (*Matricaria chamomilla* L.) is one of the most common medicinal plants in Southern and Eastern Europe. Worldwide, *Matricaria chamomilla* L. (*M. chamomilla*) is commonly known as babuna, which belongs to the family Asteraceae [14]. The chief active constituents of chamomile include phenolics, flavonoids, and phytoestrogens [15,16]. Flavonoids have been linked to several of chamomile’s pharmacological activities, which have anti-inflammatory, antioxidant, antihepatotoxic and antiviral effects along with their vasculo protective and spasmolytic impacts. The most abundant flavonoids in chamomile extract are flavon, apigenin, and luteolin. Furthermore, the alcoholic extract of *M. chamomilla* flowers is reported to reduce the histological features of PCOS in the ovary and assist luteinizing hormone (LH) excretion in rats [17].

This work relies on the assumption that if the chamomile extract has the potential to improve PCOS-related hormonal and pathological changes, it should improve the thyroid dysfunction associated with this syndrome.

The purpose of this study was to investigate the possible protective role of *M. chamomilla* flowers extract against estradiol valerate-induced hypothyroidism during PCOS. In addition, the possible antioxidant and antiapoptotic mechanisms are examined.

## 2. Materials and Methods

### 2.1. Chemicals

Estradiol valerate (purity > 99%) (ab120657), was purchased from abcam Inc, San Fran, USA. Metformin was purchased from Sigma-Aldrich Co, St Louis, MO, USA. *M. chamomilla* was purchased from World of Herbs, Assiut, Egypt. It was identified and analyzed by Analytical Chemistry Unit, Assiut University, Assiut, Egypt.

### 2.2. Preparation of Ethanolic Extract of M. Chamomilla

The powdered flowers of *M. chamomilla* were repeatedly extracted with 70% ethanol after which the solution was filtered and evaporated under vacuum to yield the extract powder.

### 2.3. Characterization of M. Chamomilla Extract Volatile Compounds

The solid phase extraction-gas chromatography/mass spectrometry (SPE-GC/MS) analysis was conducted following the previously described method [18] at the Analytical Chemistry Unit, Assiut University, Assiut, Egypt.

### 2.4. Animals

Twenty-four adult virgin female Wistar rats weighing 186 to 212 g were collected from King Fahad Medical Research Centre animal house, KAU, Jeddah, SA. The rats were left to acclimatize for 7 days at 21 °C temperature, 38% humidity, and 12:12 h light/dark cycle. There were no restrictions on feed and water offered to the rats. The research design was confirmed from the biomedical ethics research committee, college of medicine, KAU, Jeddah, SA under number (168–19).

### 2.5. Induction of PCOS and Hypothyroidism

PCOS was induced in 18 rats by injecting two estradiol valerate doses of 0.2 mg each, one dose at the beginning and the other after 6 weeks. After 6 weeks of the second estradiol valerate dose, PCOS and the associated hypothyroidism were assessed histologically and biochemically respectively. This model was previously reported by [17] and modified in our laboratory [11]. The 18 rats with PCOS were then divided into 3 groups (Groups 2, 3, and 4).

### 2.6. Study Groups

Four groups of rats were used (n = 6). Group 1: control, rats in this group were injected with 0.2 mL of corn oil. Group 2: PCOS, rats in this group were left without treatment. Group 3: *M. chamomilla*, rats in this group were orally administered *M. chamomilla* flower extract (75 mg/kg) daily for 30 days after the establishment of the model (Farideh et al. 2010). Group 4: metformin, rats in this group were orally administered metformin (50 mg/100 g body weight) daily for 30 days after the establishment of the model (Elia 2006).

### 2.7. Assessment of Percent Body Weight (% BW) Increase

Rats BW was assessed at the beginning of the experiment (initial BW) and at the end of 12 weeks (final BW). The % BW increase was calculated by the following equation:

% BW increase = ((Initial BW−Final BW)/Initial BW) × 100

### 2.8. Sampling

At the end of the experiment blood samples were gathered by heart puncture and the serum was then separated and kept frozen at −80 °C for determination of thyroid function markers and oxidative stress/antioxidant measures. The ovaries and left thyroid lobes were then dissected and kept in 10% neutral buffered formalin for assessment of PCOS induction, thyroid gland histopathological alterations and thyroid gland immunohistochemical expressions. The right thyroid lobes were collected and kept 1 h in 2.5% glutaraldehyde, postfixed for 30 min in 1% osmium tetroxide [19].

### 2.9. Assessment of Thyroid Gland Weight (Thy W)

Thy W in g was determined for each rat.

### 2.10. Assessment of PCOS Induction

Haematoxylin and eosin (H & E) stained sections of the ovary were examined for the presence of multiple cysts in the PCOS rats (n = 18) compared to the control rats (n = 6).

### 2.11. Assessment of Thyroid Function Markers

Serum TSH, triiodothyronine (T3) and T4 were measured in El-Safwa Laboratory, Tanta, Egypt, utilizing ADVIA Centaur automated competitive chemiluminescence immunoassay (Bayer HealthCare).

### 2.12. Assessment of Histopathological Alteration Using Light and Electron Microscope

For the light microscopic examination, the formalin-fixed thyroid glands were embedded in paraffin wax, serially sectioned at 3–5 mm and stained with H & E. In addition, the glutaraldehyde-fixed thyroid glands were embedded in Epon, cut at 0.5–1 mm, stained with toluidine blue.

For the electron microscope examination, ultrathin sections of the thyroid gland were stained with uranyl acetate and lead citrate.

### 2.13. Assessment of Caspase-3 and Proliferating Cell Nuclear Antigen (PCNA)Immunoexpression

Immunohistochemical staining was done utilizing the immunoperoxidase (PAP, peroxidase/antiperoxidase) technique using Lab Vision (Fremont, CA, USA) standard anti PCNA and anti-caspase-3 antibodies at 1/100 dilution. Quantification was done using ImageJ bundled with 64-bit Java 1.8.0_112, National Institute of Health, USA.

### 2.14. Assessment of Oxidative Stress/Antioxidant Measures

Serum malondialdehyde (MDA), reduced glutathione (GSH), glutathione peroxidase enzyme (GPx), catalase (CAT), and superoxide dismutase enzyme (SOD) were measured using the kits of Biodiagnostic Egypt.

### 2.15. Statistical Study

Results were represented as mean ± SE. Data were examined using ANOVA followed by the Tukey HSD test via the online calculator, astatsa.com/One-Way_Anova_with_TukeyHSD/. The *p*-value was fixed at 0.05.

## 3. Results

### 3.1. Volatile Constituents of M. Chamomilla Extract

**Figure 1:**
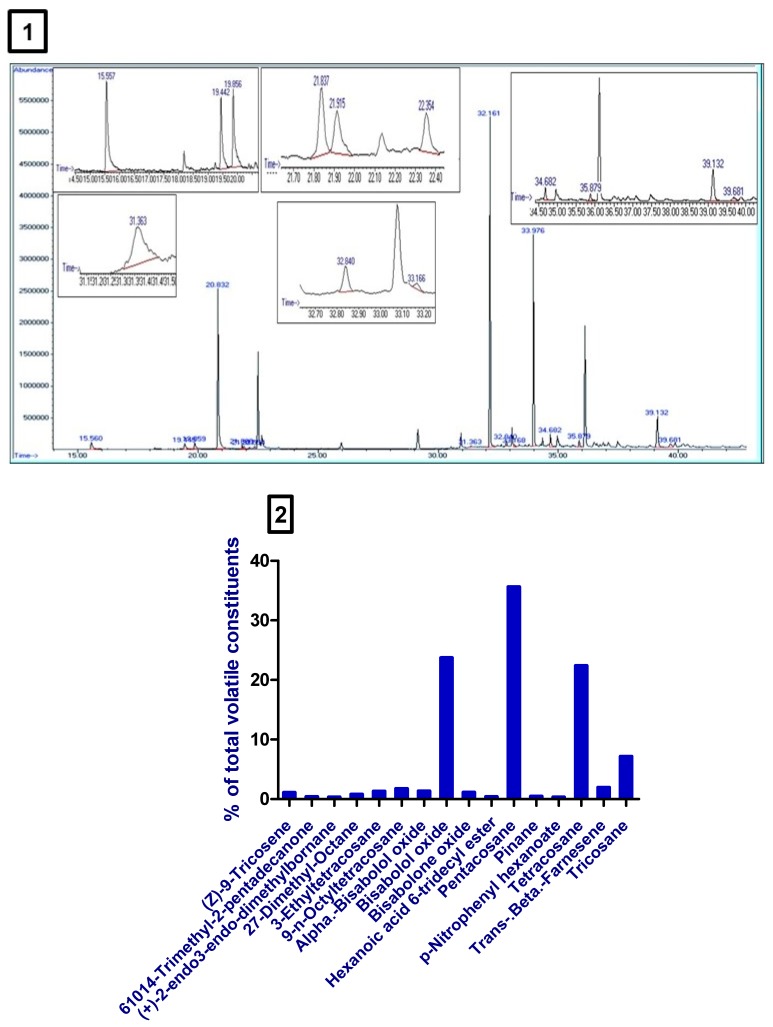
(**1**) Chromatogram obtained from SPE-GC/MS of *Matricaria chamomilla* L. (*M. chamomilla*). (**2**) A bar chart showed the % of volatile constituents present in *M. chamomilla* extract.

SPE-GC/MS analysis of *M. chamomilla* extract revealed the presence of many volatile compounds. The high concentrations compounds were bisabolol oxide, pentacosane, tetracosane, and tricosane (Table 1, Figure 1 1,2).

### 3.2. Proof of PCOS Induction

**Figure 2:**
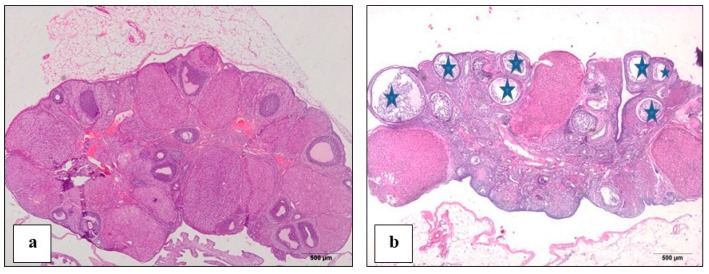
A photomicrograph (H & E) showed (**a**): control rat ovary showing normal follicles; (**b**): polycystic ovary syndrome (PCOS) rat ovary showing multiple cysts (star).

In estradiol valerate injected rats (PCOS), ovaries sections stained with H & E showed marked histological features of PCOS such as the presence of many cysts and degenerated follicles compared to the control rats’ sections (Figure 2a,b).

### 3.3. Effect of M. Chamomilla Extract and Metformin on Body and Thyroid Gland Weight

Induction of PCOS, as well as administration of either *M. chamomilla* or metformin, resulted in no changes in rats’ body weight mass (Table 2).

On the other hand, the induction of PCOS significantly (*p* < 0.01) increased rats’ thyroid gland weight relative to the control rats. Administration of *M. chamomilla* extract to PCOS rats resulted in non-significant (*p* = 0.879) changes in rats’ thyroid gland weight relative to the PCOS rats. However, the administration of metformin to PCOS rats significantly (*p* < 0.05) decreased rats’ thyroid gland weight relative to the PCOS rats (Table 2).

### 3.4. Effect of M. Chamomilla and Metformin on PCOS-Associated Thyroid Dysfunction

The induction of PCOS significantly (*p* < 0.01) increased serum TSH concentration compared to the control rats. Administration of *M. chamomilla* extract and metformin to PCOS rats resulted in a significant (*p* < 0.01) decrease in serum TSH concentration compared to the PCOS rats (Table 3).

Induction of PCOS significantly (*p* < 0.05) decreased serum T3 and T4 hormone concentration compared to the control rats. Administration of *M. chamomilla* extract and metformin to PCOS rats resulted in a significant (*p* < 0.05) increase in serum T3 and T4 hormone concentration relative to the PCOS rats (Table 3).

### 3.5. Effect of M. Chamomilla and Metformin on PCOS-Associated Increased Estrogen Level

**Figure 3:**
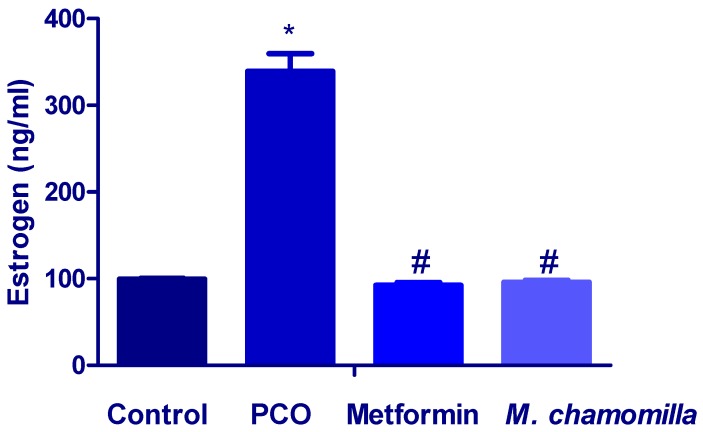
A bar chart showed the effect of *Matricaria chamomilla* L. (*M. chamomilla*) extract and metformin on serum estrogen measured in an estradiol valerate-induced polycystic ovary (PCOS) model in rats. Results are expressed as mean ± SE (n of 6 animals). The level of significance was fixed at *p* ≤ 0.05. * significant difference from the control. ^#^ significant difference from the PCOS.

The serum estrogen level was significantly (*p* < 0.01) increased in PCOS rats compared to the control rats (Figure 3). Administration of both *M. chamomilla* extract and metformin to PCOS rats resulted in a significant (*p* < 0.01) decrease in serum estrogen levels compared to the PCOS rats (Figure 3).

### 3.6. Effect of M. Chamomilla and Metformin on PCOS-Associated Thyroid Gland Histopathological Changes (H & E)

**Figure 4:**
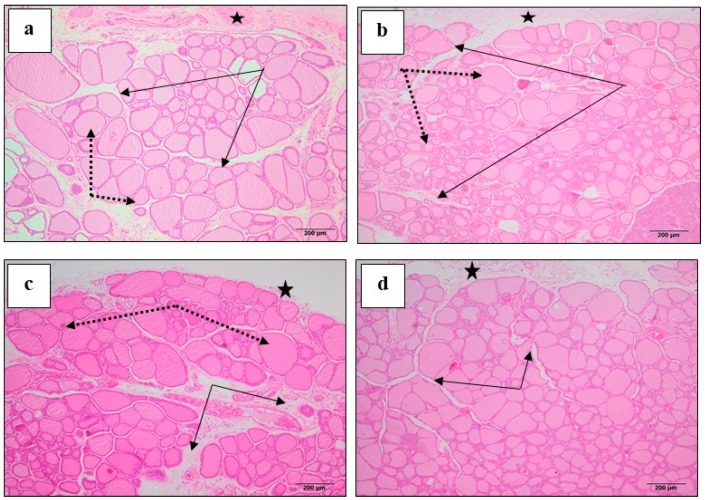
A photomicrograph (H & E ×100) showed the effect of *Matricaria chamomilla* L. (*M. chamomilla*) extract and metformin on thyroid tissue histopathological changes examined in an estradiol valerate-induced polycystic ovary (PCOS) model in rats. (**a**) The control showed connective tissue capsule (black stars), thyroid lobule separated by loose connective tissue spaces (black arrows) and thyroid follicles of various sizes (dotted arrows); (**b**) PCOS showed capsule (star), thyroid follicles (dotted arrows) are highly crowded leaving no spaces between (black arrows) and the small follicles are dominating; (**c**) *M. chamomilla* showed thyroid lobulation similar to those of the control rat; (**d**) metformin showed crowded dominating smaller-sized follicles.

**Figure 5:**
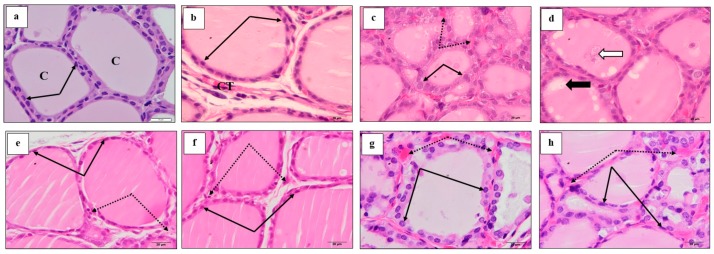
A photomicrograph (H & E ×1000) showed the effect of *Matricaria chamomilla* L. (*M. chamomilla*) extract and metformin on thyroid tissue histopathological changes examined in an estradiol valerate-induced polycystic ovary (PCOS) model in rats. (**a**,**b**) The control, (**a**) showed small and intermediate follicles lined by cells with small height (black arrows) and filled with colloid (**c**). (**b**) Showed larger follicles lined by flat epithelium. Connective tissue (CT) between follicles showed few capillaries and cells. (**c**,**d**) PCOS, (**c**) showed small dominating follicles lined by cells with more height compared to control (black arrow). CT between follicles showed dilated and congested blood capillaries (dotted arrow). (**d**) Showed some follicles with vacuoles (black arrow) and desquamated cells (white arrow) within the colloid. (**e**,**f**) *M. chamomilla* showed follicles with more or less normal histology similar to control. (**g**,**h**) Metformin showed dominating smaller follicles, most having high epithelium with active nuclei. Blood capillaries among follicles are still dilated and congested.

Control rats showed normal thyroid gland histology, where the follicles are lined by cuboidal epithelium enclosing normal luminal colloid (Figure 4a). The connective tissue between follicles showed few capillaries and cells (Figure 5a,b). The induction of PCOS resulted in numerous small follicles lined by tall large cells (Figure 4b). Follicles with damaged epithelium showed colloidal vacuolation and desquamated cells. Furthermore, the connective tissue between follicles showed increased cells and capillaries, which are dilated and congested (Figure 5c,d). Administration of *M. chamomilla* extract restored thyroid follicles histology to normal appearance, as observed in control rats (Figure 4c) and (Figure 5e,f). On the other hand, metformin administration resulted in crowded dominating smaller-sized follicles, with larger follicular cells possessing large active nuclei (Figure 4d). Blood capillaries among follicles are still dilated and congested (Figure 5g,h).

### 3.7. Effect of M. Chamomilla and Metformin on PCOS-Associated Thyroid Gland Ultrastructure Changes (Semithin Sections (Toluidine Blue) and Ultrathin Sections)

**Figure 6:**
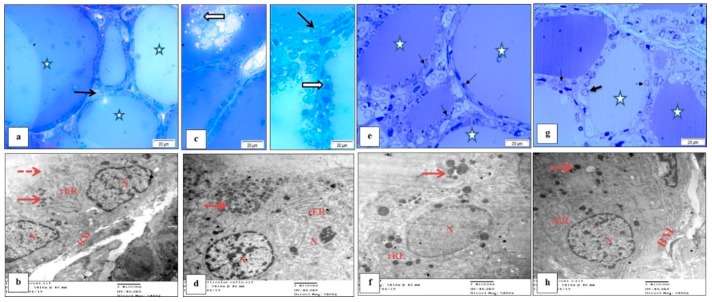
A photomicrograph showed the effect of *Matricaria chamomilla* L. (*M. chamomilla*) extract and metformin on thyroid tissue semithin sections (toluidine blue) (**a**,**c**,**e**,**g**) and ultrathin sections (**b**,**d**,**f**,**h**) changes examined in an estradiol valerate-induced polycystic ovary (PCOS) model in rats. (**a**,**b**) control, (**a**) showed follicles of various sizes, most with oval or rounded nuclei (arrow) and the lumen containing homogenous colloid (star). (**b**) Showed cell basement membrane (BM), few apical microvilli (dotted arrow), nuclei (N), rough endoplasmic reticulum (rER) and few dense granules (arrows). (**c**,**d**) PCOS, (**c**) showed swollen cells (arrows) with nuclei showing signs of karyolysis (nuclei changes of necrosis). The lumen contained cleared colloid with cell debris and numerous desquamated swollen cells with degenerated nuclei (white arrows). The photo showed another field from PCOS rat thyroid presenting 3 adjacent follicles. (**d**) Showed dilation of rER and accumulation of dense granules (arrow), part of the dense nucleus (N), and increased heterochromatin. (**e**,**f**) *M. chamomilla*, (**e**) showed nearly normal follicles with homogenous colloid free of cell debris (star). Follicular cells were similar to those of the control. Notice: the increased number of light or clear cells. (**f**) showed follicular cell with an active euchromatic nucleus (N), normal non dilated rER and few dense apical granules (arrow). (**g**,**h**) Metformin, (**g**) showed thyroid follicle with various density of colloid (star) and normal appearance of follicular cells (arrows). (**h**) Showed normal large nucleus (N), basement membrane (BM), normal rER and few dense granules.

Follicular cells of control rat thyroid glands showed active euchromatic nuclei and cell organelles including rough endoplasmic reticulum (rER) and mitochondria with normal density and dense colloidal granules (Figure 6a,b). Induction of PCOS altered normal follicular cell’s ultrastructure. Deformed nuclei with increased heterochromatin, decreased rER which looked dilated and increased dense cytoplasmic colloidal granules were observed. In addition, follicles filled with colloid contained cell debris and numerous desquamated swollen cells could be observed in semithin sections (Figure 6c,d). Semithin sections of *M. chamomilla* extract thyroid gland showed nearly normal follicles with homogenous colloid free of cell debris. Follicular cells were similar to those of the controls with an active euchromatic nucleus and normal non-dilated rER (Figure 6e,f). Furthermore, metformin administration showed a similar effect, where follicular cells appeared normal, having large nuclei and normal rER with few dense granules (Figure 6g,h).

### 3.8. Effect of M. Chamomilla and Metformin on PCOS-Associated Thyroid Gland Caspase-3 and PCNA Immunoexpression

**Figure 7:**
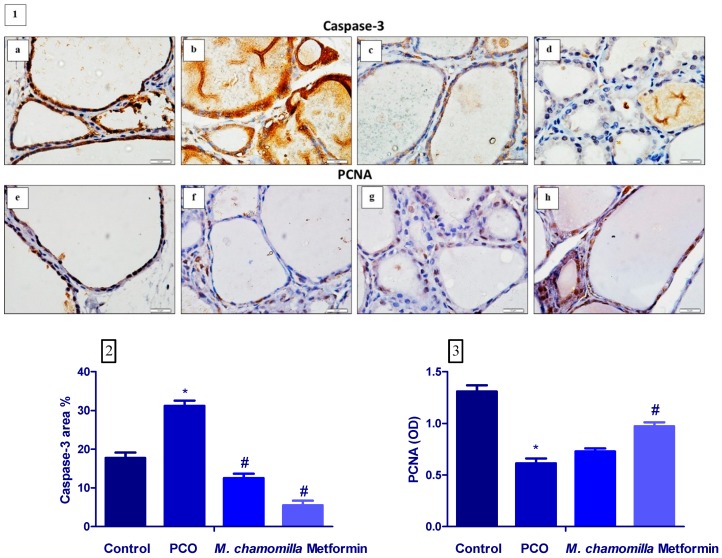
(**1**) A photomicrograph showed the effect of *Matricaria chamomilla* L. (*M. chamomilla*) extract and metformin on thyroid tissue caspase-3 expression (**a**–**d**) and PCNA (**e**–**h**) examined in an estradiol valerate-induced polycystic ovary (PCOS) model in rats. (**a**,**e**) Control, (**b**,**f**) PCOS, (**c**,**g**) *M. chamomilla*, (**d**,**h**) metformin. (**2**,**3**) Bar charts showed the effect of *Matricaria chamomilla* L. (*M. chamomilla*) extract and metformin on thyroid tissue caspase-3 (area %) and PCNA (OD). Results are expressed as mean ± SE (n of 6 animals). The level of significance was fixed at *p* ≤ 0.05. * significant from the control group. ^#^ significant from the PCOS group.

Induction of PCOS significantly (*p* < 0.01) increased thyroid gland caspase-3 expression as compared to the control rats. Administration of *M. chamomilla* extract and metformin to PCOS rats resulted in a significant decrease in thyroid gland caspase-3 expression compared to the PCOS rats (Figure 7 1,2).

The induction of PCOS significantly (*p* < 0.01) decreased thyroid gland PCNA expression compared to the control rats. Administration of *M. chamomilla* extract to PCOS rats resulted in a non-significant change in thyroid gland PCNA expression as compared to the PCOS rats. On the other hand, the administration of metformin extract to PCOS rats significantly (*p* < 0.01) increased thyroid gland PCNA expression compared to the PCOS rats (Figure 7 1,3).

### 3.9. Effect of M. Chamomilla and Metformin on PCOS-Associated Oxidative Stress Status

**Figure 8:**
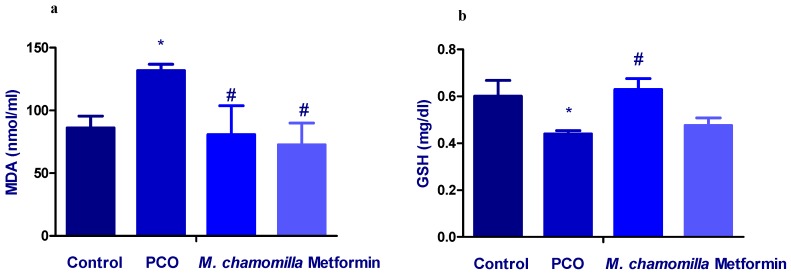
A bar chart showed the effect of *Matricaria chamomilla* L. (*M. chamomilla*) extract and metformin on serum (**a**) MDA and (**b**) GSH measured in an estradiol valerate-induced polycystic ovary (PCOS) model in rats. Results are expressed as mean ± SE (n of 6 animals). The level of significance was fixed at *p* ≤ 0.05. * significant difference from the control. ^#^ significant difference from the PCOS.

The induction of PCOS significantly (*p* < 0.01) increased serum MDA concentration compared to the control rats. Administration of *M. chamomilla* extract and metformin to PCOS rats resulted in a significant (*p* < 0.05) decrease in serum MDA concentration compared to the PCOS rats (Figure 8a).

The induction of PCOS significantly (*p* < 0.05) decreased serum GSH concentration compared to the control rats. Administration of *M. chamomilla* extract to PCOS rats resulted in a significant (*p* < 0.01) increase in serum GSH concentration compared to the PCOS rats. On the other hand, the administration of metformin to PCOS rats resulted in a non-significant change in serum GSH concentration compared to the PCOS rats (Figure 8b).

Induction of PCOS significantly (*p* < 0.01) decreased serum GPx and CAT concentration compared to the control rats. Administration of *M. chamomilla* extract and metformin to PCOS rats resulted in significant (*p* < 0.01) increases in serum GPx and CAT concentration compared to the PCOS rats. On the other hand, the serum concentration of SOD showed no significant changes across all the experimental groups (Table 4).

## 4. Discussion

This study clearly showed that the ethanolic extracts of *M. chamomilla* relieved the thyroid gland hypofunction demonstrated in estradiol valerate-induced PCOS in rats after 30 days of treatment. This therapeutic effect was characterized by restored serum TSH and estrogen levels, increased serum T3 and T4 levels, reduced oxidative stress, and improved antioxidant capacity. The thyroid gland histological study also showed significant improvements in the histology of thyroid follicles. Furthermore, *M. chamomilla* extract significantly reduced the apoptosis of the thyroid gland.

Several researchers have established an increased incidence of thyroid autoimmunity in females with PCOS. A previous study demonstrated higher concentrations of thyroid antibody in females with PCOS, increased thyroid size and increased incidence of hypoechogenic thyroid glands (favorable with thyroiditis). Moreover, in the same research, PCOS females showed an increased TSH concentration [20]. Likewise, this study showed increased thyroid gland weight and decreased serum TSH in PCOS rats suggesting that it may be autoimmune thyroiditis accompanying PCOS caused by estradiol valerate. So far, the pathophysiological mechanism that connects PCOS to hypothyroidism has not been strictly defined. Currently, the best explanation seems to be overlapping pathways with a complex interplay between PCOS, fattiness, thyroid hypofunction, and autoimmunity, operating to generate convergent clinical images [21]. A reasonable elucidation for higher thyroid autoimmunity in PCOS is probably due to the hyperestrogenic status of PCOS. Hyperestrogenism was suggested as a reason for enhanced autoimmune disorders in females contrasted with males [22,23]. Similarly, the results of this study showed increased serum concentration of estrogen in rats in the PCOS group together with the increased level of serum TSH, decreased T3 and T4 serum levels.

Treatment of PCOS rats with *M. chamomilla* extract significantly ameliorated the hypothyroid function as confirmed by the improved thyroid hormones and histological structure of thyroid follicles. Moreover, caspase-3 (apoptotic marker) expression was decreased in the thyroid gland of *M. chamomilla* rats compared to PCOS rats. This may be due to *M. chamomilla* caused a drop in the level of serum estrogen [15]. Phytoestrogens regulate the function of aromatase and the activity of major enzymes in estrogen biosynthesis. In the ovary, the enzyme converts androgens into estrogens. Thus, as phytoestrogens inhibit this enzyme, this process is blocked, thereby reducing the concentration of estrogen [24]. Chamomile extract constitutes the phytoestrogen active constituents; coumarins, which may decrease estrogen formation [25]. Furthermore, phytoestrogens inhibit the function of cytochrome P450 enzymes, thereby hampering cholesterol conversion to pregnenolone, and thus decreasing the production of estrogen [26,27]. In addition, the increase in free T3 and T4 is responsible for the decrease in TSH hormone.

Treatment of PCOS rats with metformin significantly ameliorated the hypothyroid function as confirmed by the decreased TSH and improved T3 and T4 hormones relative to PCOS rats. The results were in agreement with those of Xiaowen et al. [28], who reported that that metformin may directly affect the synthesis of thyroid hormone and then decrease the level of TSH. It has been demonstrated that metformin crosses the blood-brain barrier and a central mechanism of TSH inhibition may thus be an attractive explanation. Even though it activates AMPK in the periphery, metformin suppresses AMPK activity in the hypothalamus and possibly counteracts hypothalamic T3 action on TSH secretion [29].

Also, the caspase-3 expression was decreased in *M. chamomilla* rats compared to PCOS rats. These histological features were generally correlated to the thyroidal functional status because the serum T3 and T4 levels were enhanced in animals treated with estrogen or tamoxifen as compared to the vehicle-treated controls.

Oxidative stress occurs when the generation of reactive oxygen species (ROS) exceeds the activity of the antioxidant molecules and enzymes, resulting in injurious consequences for the body tissues and cells [30]. Studies have reported that PCOS was accompanied by oxidative stress that may result from obesity, hyperinsulinemia, and dyslipidemia. Administration of many antioxidants like omega-3 fatty acids, α-lipoic acid, and N-acetylcysteine ameliorated hyperlipidemia and insulin resistance in PCOS females [31]. Likewise, this study’s results showed increased serum MDA and decreased the serum concentration of GSH, GPx, and CAT in PCOS rats. Treatment of PCOS rats with *M. chamomilla* extract significantly improved serum oxidant/antioxidant status, compared to PCOS rats, which may be correlated with the improved PCOS-associated thyroid gland hypofunction. Chamomile contains plentiful antioxidants’ active constituents [32]. This study showed that the volatile chemical ingredients of *M. chamomilla* consist of sesquiterpenes compounds (α-bisabolol, bisabolol oxides, bisabolone oxide, trans-beta-farnesene), pentacosane, tetracosane, and tricosane [33,34,35]. The sesquiterpenes compounds may be essential for the antioxidant function of *M. chamomilla* extract [36,37].

## 5. Conclusions

The histological examination of the thyroid gland with both light and electron microscopy, as well as the hormone measurements, showed the efficacy of *M. chamomilla* ethanolic extract and metformin in improving thyroid hypofunction associated with PCOS in rats, through an antioxidant mechanism.

## Figures and Tables

**Table 1:** Volatile constituents of *Matricaria chamomilla* L. (*M. chamomilla*) extract detected by solid phase extraction-gas chromatography/mass spectrometry (SPE-GC/MS).

Serial Number	Retention Time (min)	Compound Name	Relative Area (%)
1	35.88	(Z)-9-tricosene	0.67
2	21.915	6,10,14-trimethyl-2-pentadecanone	0.24
3	22.358	(+)-2-endo,3-endo-dimethylbornane	0.19
4	32.844	2,7-dimethyl-octane	0.48
5	39.681	3-ethyltetracosane	0.81
6	34.68	9-n-octyltetracosane	1.08
7	19.444	Alpha-bisabolol oxide	0.84
8	20.831	Bisabolol oxide	14.69
9	19.858	Bisabolone oxide	0.70
10	31.363	Hexanoic acid, 6-tridecyl ester	0.23
11	32.162	Pentacosane	22.05
12	21.839	Pinane	0.27
13	33.17	p-Nitrophenyl hexanoate	0.19
14	33.98	Tetracosane	13.86
15	15.562	Trans-beta-farnesene	1.20
16	39.133	Tricosane	4.43

**Table 2:** Effect of *Matricaria chamomilla* L. (*M. chamomilla*) extract and metformin on percent body weight (% BW) increase and thyroid gland weight (Thy W) measured in an estradiol valerate-induced polycystic ovary (PCOS) model in rats.

	% BW Increase	Thy W (mg)
Control	15.79 ± 2.1	4.14 ± 1.7
PCOS	14.34 ± 2.0	29.16 ± 2.2 ^a^
*M. chamomilla*	18.4 ± 2.0	32.05 ± 2.8
Metformin	10.68 ± 2.1	12.23 ± 4.3 ^b^

Results are expressed as mean ± SE (n of 6 animals). The level of significance was fixed at *p* ≤ 0.05. ^a^ significant from the control group. ^b^ significant from the PCOS group.

**Table 3:** Effect of *Matricaria chamomilla* L. (*M. chamomilla*) extract and metformin on serum thyroid function markers measured in an estradiol valerate-induced polycystic ovary (PCOS) model in rats.

	Control	PCOS	*M. chamomilla*	Metformin
**TSH (μU/mL)**	0.012 ± 0.002	0.089 ± 0.027 ^a^	0.013 ± 0.003 ^b^	0.018 ± 0.005 ^b^
**T3 (pg/mL)**	2.68 ± 0.17	2.13 ± 0.12 ^a^	2.62 ± 0.14 ^b^	2.67 ± 0.11 ^b^
**T4 (ng/dL)**	2.07 ± 0.10	1.59 ± 0.02 ^a^	1.96 ± 0.12 ^b^	2.38 ± 0.16 ^b^

Results are expressed as mean ± SE (n of 6 animals). The level of significance was fixed at *p* ≤ 0.05. ^a^ significant from the control group. ^b^ significant from the PCOS group. ANOVA was adopted. TSH: thyroid stimulating hormone; T3: triiodothyronine; T4: thyroxine.

**Table 4:** Effect of *Matricaria chamomilla* L. (*M. chamomilla*) extract and metformin on serum antioxidant enzymes measured in an estradiol valerate-induced polycystic ovary (PCOS) model in rats.

	Control	PCOS	*M. chamomilla*	Metformin
**GPx (mU/mL)**	23.0 ± 1.7	14.9 ± 1.7 ^a^	26.1 ± 2.5 ^b^	23.0 ± 2.5 ^b^
**CAT (U/mL)**	321 ± 35	148 ± 1.8 ^a^	292 ± 1.2 ^b^	248 ± 3.2 ^b^
**SOD (U/mL)**	345 ± 19	335 ± 7.2	344 ± 28	327 ± 16

Results are expressed as mean ± SE (n of 6 animals). The level of significance was fixed at *p* ≤ 0.05. ^a^ significant from the control group. ^b^ significant from the PCOS group. ANOVA was adopted. GPx: glutathione peroxidase; CAT: catalase; SOD: superoxide dismutase.

## Abbreviations

SCH: Subclinical hypothyroidism; TSH: thyroid-stimulating hormone; T4: Thyroxine; PCOS: Polycystic ovarian syndrome; *M. chamomilla*: *Matricaria chamomilla* L; LH: luteinizing hormone; SPE-GC/MS: solid phase extraction-gas chromatography/mass spectrometry; % BW: Percent body weight; Thy W: thyroid gland weight; H & E: Haematoxylin and eosin; T3: Triiodothyronine; PAP: peroxidase/antiperoxidase; MDA: Malondialdehyde; GSH: Reduced glutathione; GPx: Glutathione peroxidase; CAT: Catalase; SOD: Superoxide dismutase; ANOVA: Analysis of variance; rER: Endoplasmic reticulum; PCNA: proliferating cell nuclear antigen; ROS: Reactive oxygen species.

## References

[1] Teng W., Shan Z., Patil-Sisodia K., Cooper D.S. (2013). Hypothyroidism in pregnancy. Lancet Diabetes Endocrinol..

[2] Cho M.K. (2015). Thyroid dysfunction and subfertility. Clin. Ex. Reproductive Med..

[3] Barišić T., Mandić V., Vasilj A., Tiric D. (2019). Higher levels of thyrotropin in pregnancy and adverse pregnancy outcomes. J. Maternal-Fetal Neonatal Med..

[4] Yang J., Liu Y., Liu H., Zheng H., Li X., Zhu L., Wang Z. (2018). Associations of maternal iodine status and thyroid function with adverse pregnancy outcomes in Henan Province of China. J. Trace Elem. Med. Biol..

[5] Ganie M.A., Laway T.A., Wani M.A., Zargar S., Nisar F., Ahamed M.L.K., Ahmed S. (2011). Association of subclinical hypothyroidism and phenotype, insulin resistance, and lipid parameters in young women with polycystic ovary syndrome. Fertil. Sterility.

[6] Celik C., Abali R., Tasdemir N., Guzel S., Yuksel A., Aksu E., Yılmaz M. (2012). Is subclinical hypothyroidism contributing dyslipidemia and insulin resistance in women with polycystic ovary syndrome?. Gynecol. Endocrinol..

[7] March W.A., Moore V.M., Willson K.J., Phillips D.I., Norman R.J., Davies M.J. (2010). The prevalence of polycystic ovary syndrome in a community sample assessed under contrasting diagnostic criteria. Hum. Reprod..

[8] Moran L.J., Norman R.J., Teede H.J. (2015). Metabolic risk in PCOS: Phenotype and adiposity impact. Trends Endocrinol. Metab..

[9] Ding X., Yang L., Tang R., Chen Q., Pan J., Yang H., Chen Z., Chen X., Mu L. (2018). Subclinical Hypothyroidism in Polycystic Ovary Syndrome: A Systematic Review and Meta-Analysis. Front. Endocrinol..

[10] Duran C., Basaran M., Kutlu O., Kucukaydin Z., Bakdik S., Burnik F.S., Aslan U., Erdem S.S., Ecirli S. (2015). Frequency of nodular goiter and autoimmune thyroid disease in patients with polycystic ovary syndrome. Endocrine.

[11] Alzahrani A.A., Alahmadi A.A., Ali S.S., Alahmadi B.A., Arab R.A., Wahman L.F., El-Shitany N.A. (2019). Biochemical and histological evidence of thyroid gland dysfunction in estradiol valerate model of the polycystic ovary in Wistar rats. Biochem. Biophys. Res. Commun..

[12] Singh K.B., Michael Conn P. (2008). Rat models of polycystic ovary syndrome. Sourcebook of Models for Biomedical Research.

[13] Jelodar G., Masoomi S., Rahmanifar F. (2018). Hydroalcoholic extract of flaxseed improves polycystic ovary syndrome in a rat model. Iran. J. Basic Med. Sci..

[14] Singh O., Khanam Z., Misra N., Srivastava M.K. (2011). Chamomile (*Matricaria chamomilla* L.): An overview. Pharmacogn. Rev..

[15] Johari H., Sharifi E., Mardan M., Kafilzadeh F., Hemayatkhah V., Kargar H., Nikpoor N. (2011). The effects of a hydroalcoholic extract of *Matricaria chamomilla* flower on the pituitary-gonadal axis and ovaries of rats. Int. J. Endocrinol. Metab..

[16] Heidary M., Yazdanpanahi Z., Dabbaghmanesh M.H., Parsanezhad M.E., Emamghoreishi M., Akbarzadeh M. (2018). Effect of chamomile capsule on lipid- and hormonal-related parameters among women of reproductive age with polycystic ovary syndrome. J. Res. Med. Sci..

[17] Zangeneh F.Z., Minaee B., Amirzargar A., Ahangarpour A., Mousavizadeh K. (2010). Effects of chamomile extract on biochemical and clinical parameters in a rat model of polycystic ovary syndrome. J. Reprod. Infertility.

[18] Neamatallah T., El-Shitany N.A., Abbas A.T., Ali S.S., Eid B.G. (2018). Honey protects against cisplatin-induced hepatic and renal toxicity through inhibition of NF-κB-mediated COX-2 expression and the oxidative stress dependent BAX/Bcl-2/caspase-3 apoptotic pathway. Food Funct..

[19] Sobota J.A., Bäck N., Eipper B.A., Mains R.E. (2009). Inhibitors of the V0 subunit of the vacuolar H+-ATPase prevent segregation of lysosomal- and secretory-pathway proteins. J. Cell Sci..

[20] Janssen O.E., Mehlmauer N., Hahn S., Offner A.H., Gartner R. (2004). High prevalence of autoimmune thyroiditis in patients with polycystic ovary syndrome. Eur. J. Endocrinol..

[21] Goyal D., Relia P., Sehra A., Khandelwal D., Dutta D., Jain D., Kalra S. (2019). Prevalence of hypothyroidism and thyroid autoimmunity in polycystic ovarian syndrome patients: A North Indian study. Thyroid Res. Pract..

[22] Fénichel P., Gobert B., Carré Y., Barbarino-Monnier P., Hiéronimus S. (1999). Polycystic ovary syndrome in autoimmune disease. Lancet.

[23] Reimand K., Talja I., Metsküla K., Kadastik Ü., Matt K., Uibo R. (2001). Autoantibody studies of female patients with reproductive failure. J. Reproductive Immunol..

[24] Chen S., Cho M., Karlsberg K., Zhou D., Yuan Y.C. (2004). Biochemical and biological characterization of a novel anti-aromatase coumarin derivative. J. Biol. Chem..

[25] Brueggemeier R.W., Gu X., Mobley J.A., Joomprabutra S., Bhat A.S., Whetstone J.L. (2001). Effects of Phytoestrogens and Synthetic Combinatorial Libraries on Aromatase, Estrogen Biosynthesis, and Metabolism. Ann. N. Y. Acad. Sci..

[26] Löfgren S., Hagbjörk A.L., Ekman S., Fransson-Steen R., Terelius Y. (2004). Metabolism of human cytochrome P450 marker substrates in mouse: A strain and gender comparison. Xenobiotica.

[27] Ronis M.J. (2016). Effects of soy containing diet and isoflavones on cytochrome P450 enzyme expression and activity. Drug Metab. Rev..

[28] Hu X., Liu Y., Wang C., Hou L., Zheng X., Xu Y., Ding L., Pang S. (2017). Metformin affects thyroid function in male rats. Oncotarget.

[29] Pappa T., Alevizaki M. (2013). Metformin and Thyroid: An Update. Eur. Thyroid J..

[30] Mohammadi M. (2019). Oxidative stress and polycystic ovary syndrome: A brief review. Int. J. Preventive Med..

[31] Macut D., Bjekić-Macut J., Savić-Radojević A. (2013). Dyslipidemia and oxidative stress in PCOS. Polycystic Ovary Syndrome.

[32] Bhaskaran N., Shukla S., Kanwal R., Srivastava J.K., Gupta S. (2012). Induction of heme oxygenase-1 by chamomile protects murine macrophages against oxidative stress. Life Sci..

[33] Srivastava J.K., Gupta S. (2009). Extraction, characterization, stability and biological activity of flavonoids isolated from chamomile flowers. Mol. Cell. Pharmacol..

[34] Ganzera M., Schneider P., Stuppner H. (2006). Inhibitory effects of the essential oil of chamomile (*Matricaria recutita* L.) and its major constituents on human cytochrome P450 enzymes. Life Sci..

[35] Lopez M., Blazquez M.A. (2016). Characterization of the Essential Oils from Commercial Chamomile Flowers and Chamomile Teabags by GC-MS Analysis. Int. J. Pharmacogn. Phytochem. Res..

[36] Fırat Z., Demirci F., Demirci B. (2018). Antioxidant activity of chamomile essential oil and main components. Nat. Volatiles Essent. Oils.

[37] Khayyal M.T., Kreuter M.H., Kemmler M., Altmann P., Abdel-Naby D.H., El-Ghazaly M.A. (2019). Effect of a chamomile extract in protecting against radiation-induced intestinal mucositis. Phytotherapy Res..

